# Real World Evidence Versus Randomised Controlled Trials: Is the Future of Nutritional Sciences Research in Electronic Health Records?

**DOI:** 10.1111/nbu.70046

**Published:** 2026-01-23

**Authors:** Kathryn V. Dalrymple

**Affiliations:** ^1^ Department of Nutritional Sciences, School of Life Course & Population Sciences King's College London London UK

**Keywords:** bias, electronic health records, generalisability, population level data, quasi‐experimental design, randomised controlled trials

## Abstract

Randomised controlled trials (RCTs) are the gold standard of research studies. They aim to recruit participants with similar characteristics and randomly assign them to a treatment or control/placebo arm. Due to randomisation, RCTs provide comprehensive, unbiased evidence about treatment efficacy and safety and examine cause‐and‐effect relationships between the intervention and outcome. However, RCTs are expensive, recruitment can be time‐consuming and high drop‐out rates can reduce internal validity. Depending on the target population, findings are not always generalisable at a population level. Of relevance to nutritional sciences, due to the type of research questions, researchers and participants cannot always be blinded to randomisation. Electronic health records (EHRs) provide a possible solution to some of these constraints. Using data from healthcare systems may help to reduce costs and overcome logistical challenges as (1) pragmatic trials integrated into routine care enable real‐time data analysis and faster translation of findings and (2) once dynamic longitudinal cohorts have been generated, they can be analysed using quasi‐experimental designs. These have the potential to provide population level data with higher generalisability, lower attrition and greater statistical power. EHRs do come with their own challenges, including the lack of a uniform information infrastructure, missing data and data quality. There are also ethical considerations, as patients may not wish for their data to be used in a research capacity, which in turn can affect the generalisability of findings. When it comes to nutritional sciences and generating evidence, there is no one‐size fits all approach. EHRs offer great potential for advancing certain research questions, such as when there is a population level intervention, for example, the soft drinks industry levy or the inclusion of folic acid in non‐wholemeal wheat flour. EHRs offer the opportunity to integrate multiple datasets which will enable a comprehensive understanding of a nutrition intervention impact on health and disease in diverse populations and real‐world settings. However, RCTs remain imperative for understanding causality. The scope of this review is to examine how RCTs and EHRs can be used to generate evidence in nutritional sciences, highlighting their respective opportunities and challenges.

## 1 Introduction

### 1.1 A Brief History of Nutrition Research and Clinical Trials

Diet and nutrition research has played its part in health research for centuries. The first documented example of clinical research dates back to 562 bc and focuses on a nutritional intervention. According to The Bible, King Nebuchadnezzar of Babylon conducted an experiment where he ordered his citizens to either eat a diet of meat and wine or legumes and water. He hypothesised that a diet high in the former would be beneficial for physical conditioning. At the end of the 10‐day experiment, the legumes and water group appeared healthier and, as a result, King Nebuchadnezzar allowed his citizens to continue their diet. In more modern times, Dr. James Lind, a naval surgeon, is considered to have undertaken the first controlled clinical trial, which addressed vitamin C deficiency. In 1747, whilst working on board HMS Salisbury, Lind conducted a comparative trial to identify an effective treatment for scurvy. Twelve sailors with scurvy symptoms were divided into six groups, each receiving one of the following interventions: (1) cyder, (2) elixir of vitriol (a mixture of sulphuric acid and alcohol), (3) vinegar, (4) sea‐water, (5) oranges and lemons, or (6) an electuary made up by Lind, which was a combination of garlic, mustard seed, horseradish, balsam of Peru and gum myrrh. Within six days, the sailors consuming oranges and lemons recovered and were assigned to care for the remaining ill participants (Lind 1753). Due to the high cost of oranges and lemons at the time, it took the Royal Navy nearly 50 years to make lemon juice a compulsory part of a sailor's diet (Dunn 1997).

### 1.2 The Modern Day Randomised Controlled Trial

In 1948 the first randomised controlled trial (RCT) was published (Marshall et al. 1948). Funded by the Medical Research Council (MRC), streptomycin and bed rest (*n* = 55) versus best rest alone (*n* = 52) were tested as treatments for tuberculosis. Participants were aged between 15 and 30 years and 40% were male. Sir Austin Bradford‐Hill, epidemiologist and author, played a pivotal role in shaping RCT methodology by introducing the concept of random allocation of participants to different treatment groups (Wilkinson 1997). The streptomycin study implemented clear inclusion criteria, random sampling (stratified by sex), a protocol for data collection and blinding of investigators; features which are common in RCT methodology today (Altman 1996; Sibbald and Roland 1998).

RCTs are widely accepted as the gold standard of nutrition research studies due to their ability to provide robust, unbiased evidence about the efficacy and safety of interventions (Hariton and Locascio 2018). They aim to recruit participants with similar characteristics and randomly assign them to 2 or more treatment arms, one of which is a control or placebo group. Randomisation is intended that, on average, the participants in different groups are comparable across characteristics such as age, gender and ethnicity. By balancing both known and unknown factors that may influence outcomes, it increases the likelihood that any differences can be attributed to the treatment rather than external factors. Randomisation is a powerful tool as it allows researchers to examine cause‐and‐effect relationships between the intervention and an outcome of interest (Pearl and Mackenzie 2018) by reducing selection and confounder biases (Coggon et al. 2003; Berger et al. 2021). These types of bias are common issues in observational data, for example, data from prospective cohort and case–control studies (Sterrantino 2024). Additionally, RCTs often incorporate blinding, where participants and/or researchers are unaware of the treatment group to which individual participants have been randomised (Higgins et al. 2011). This reduces the potential for performance and detection bias in treatment administration, data collection, analysis and reporting of results, further increasing the reliability of the findings (Mansournia et al. 2017).

To ensure ethical and scientific quality standards in the design, conduct, documentation and reporting of clinical trials are met, the SPIRIT (Standard Protocol Items: Recommendations for Interventional Trials) guidelines (SPIRIT 2013) provide a checklist to ensure that a clinical trial protocol is complete, well‐structured and clearly reported. This approach ensures greater precision during the data collection phase of the study and the results from RCTs are regarded as having high internal validity. RCTs must also adhere to ethical principles (World Medical Association 2013), and comply with Good Clinical Practice (National Institute of Health Research 2024). Also, to ensure transparency in reporting, the CONSORT (Consolidated Standards of Reporting Trials) guidelines outline how to clearly present data in publications (Altman 1996).

Once the data has been collected, there are defined approaches for its analysis and presentation. The most common approach for analysing RCT data is the intention to treat (ITT) method (Smith et al. 2021). In the ITT approach, all randomised participants are included in the analysis, based on the group to which they were initially randomly assigned. Alternative methods include: (1) as‐treated (AT): this method considers participants based on the treatment they actually received, rather than the treatment to which they were randomised. AT is useful if participants deviate from the protocol; (2) Complete case; participants with missing data are excluded from the analysis. Although the complete case approach is straightforward to analyse, there is a risk of bias if the missing data are related to the intervention or the primary outcome of interest (further details below in the missing data section); (3) Per‐protocol only participants who fully adhere to the study protocol are included in the final analysis. However, the per‐protocol approach can reduce internal validity if the study results deviate from the original randomisation schedule (Smith et al. 2021) since the group of participants who adhere to the intervention may no longer represent the original randomised cohort. For example, participants from lower socio‐economic backgrounds may face barriers to attend follow‐up appointments due to time constraints or cost; therefore, they are unable to adhere to the protocol. In contrast, those from higher socio‐economic backgrounds may not face such barriers and as a result may be over‐represented in the study population.

### 1.3 Data From Randomised Controlled Trials and Their Impact on Nutritional Research

In the field of nutritional sciences, RCTs have played an influential role in shaping dietary guidelines, developing public health interventions and understanding the impact of nutritional deficiencies on health outcomes. For example, the Dietary Approaches to Stop Hypertension clinical trial in 459 participants demonstrated that a diet rich in fruits, vegetables and low‐fat dairy foods and with reduced saturated and total fat can substantially lower blood pressure (Appel et al. 1997). This study directly influenced dietary recommendations for hypertension management, a major risk factor for coronary heart disease and ischemic or haemorrhagic stroke (Filippou et al. 2020), conditions that are currently the leading cause of death worldwide (Naghavi et al. 2024).

The MRC Vitamin Study Research Group (1991) aimed to assess the effectiveness of folic acid supplementation in reducing the risk of neural tube defects (NTDs) in 1817 high‐risk pregnancies. This RCT provided unequivocal evidence of a causal relationship between maternal folate in early pregnancy and the development of NTDs, a congenital condition which affects approximately 1000 pregnancies/year in the UK (Morris et al. 2021). The results from the MRC study have led to worldwide public health initiatives. To date, nearly 70 countries have introduced mandatory folic acid fortification of staple foods (including rice, wheat flour and maize flour) leading to a 50% lower NTD prevalence compared to those countries without fortification (Quinn et al. 2024). In the UK, following recommendations from the Scientific Advisory Committee on Nutrition (2006) and a recent public health campaign, the Government has mandated folic acid fortification of non‐wholemeal flour (0.25 mg/100 g), which is due to become effective from October 2026 (UK Government 2024). It is estimated that this level of fortification will prevent 20% of NTD cases in the UK (Mayer et al. 2017; Wald 2022).

RCTs have also provided us with evidence of how well‐intentioned interventions can yield harmful results. The Alpha‐Tocopherol Beta Carotene Cancer Prevention Study Group (1994) investigated whether supplementation with alpha‐tocopherol (vitamin E) and beta‐carotene could reduce the incidence of lung cancer and other cancers in male smokers. A total of 29 133 male smokers aged 50–69 years from Finland were randomly assigned to one of four regimens: alpha‐tocopherol (50 mg per day) alone, beta carotene (20 mg per day) alone, both alpha‐tocopherol and beta carotene, or placebo. Follow‐up continued for 5–8 years. The authors reported an 18% increase in lung cancer risk among participants taking beta‐carotene compared to those not receiving it. Others studies of high dose supplementation of vitamin C and E in pregnancy (Poston et al. 2006) have also reported potential adverse birth outcomes. These analyses provide us with evidence of the adverse effect high‐dose supplementation can have on health outcomes, emphasising that greater intake is not necessarily beneficial. They also reinforce the need for rigorously designed RCTs to validate the safety and efficacy of nutritional interventions before they are recommended for public use.

### 1.4 Limitations of Randomised Controlled Trials

Despite their proven value, RCTs do have their limitations. They can be expensive, requiring a dedicated clinical trials infrastructure that includes a research team, administrative staff and infrastructure costs for them to be managed appropriately (Hind et al. 2017). Recruitment and follow‐up of participants can also be time‐consuming; therefore, delaying research outputs. This issue is particularly prominent in pregnancy research. In the author's experience as a researcher in women's health, they have encountered challenges while working on the UK Pregnancy Better Eating and Activity Trial (UPBEAT). UPBEAT aimed to assess whether a behavioural intervention of diet and physical activity in pregnant women with obesity could reduce the risk of gestational diabetes mellitus (GDM) and large for gestational age infants (Poston et al. 2015). UPBEAT was a multi‐centre RCT conducted across eight UK inner‐city hospitals, and 1554 women were randomised in early pregnancy to the study. Recruitment, intervention delivery and follow‐up through pregnancy to inform the primary clinical outcomes lasted for over 5 years (Poston et al. 2015). Subsequent postnatal follow‐ups at 6‐months (Patel et al. 2017) and 3‐years (Dalrymple et al. 2020) extended the study timeline by an additional 5 years. As a result, the completion of the study and publication of findings for the secondary outcomes required more than 10 years. Attrition rates were high during these follow‐ups, with only 50% and 34% returning for the respective appointments, and those participants who did attend the follow‐up appointments were not representative of the original randomised cohort, with evidence suggesting that participants of higher socioeconomic status or educational attainment were more likely to participate in follow‐up assessments. This may have introduced bias and potentially diluted the findings of these follow‐ups, limiting their generalisability to the broader population. However, it could also be argued that if positive impacts of the behavioural intervention were observed within these follow‐up groups, the potential for benefit across the entire UPBEAT cohort may be even greater. These challenges highlight the realities of conducting long‐term follow‐up in RCTs, and align with broader concerns in the literature regarding the impact of attrition on the robustness of their findings. These challenges illustrate how the complex logistics of RCTs can delay and impact on critical findings. Despite these difficulties, UPBEAT reported important findings; it was the first RCT in pregnancy to demonstrate that a behavioural intervention in pregnancy has the potential to reduce infant adiposity at 6‐months of age (Patel et al. 2017), improve cardiovascular function at 3‐years of age (Dalrymple et al. 2020) and sustain improvements in maternal diet up to 3‐years after delivery (Dalrymple et al. 2020).

Unless there is clinical equipoise, where there is uncertainty about the superiority of one treatment over another (Freedman 1987), the inclusion of a control or placebo group in RCTs may raise ethical concerns. This is particularly relevant when participants in the control arm are denied access to an intervention that may already be known to be effective. In some areas of clinical practice, such as oncology research, denying potentially life‐saving treatments may raise significant ethical dilemmas (Nardini 2014). Furthermore, depending on the nature of the intervention, it may not always be feasible to blind researchers, clinicians, or participants to the randomisation process, which can introduce potential bias and affect the validity of the study. In nutritional sciences, double‐blind placebo RCTs are common in nutrient supplement trials (Wood et al. 2023); however, blinding of participants can become challenging in whole‐diet intervention (Staudacher et al. 2022), thus reducing the validity of the results. Alternative study designs, such as the step‐wedge trial (Ellenberg 2018), may help to mitigate the problems associated with blinding. In a step‐wedge design, clusters start as controls, and the intervention is rolled out to different clusters at random over time until all receive it, allowing both control and intervention data to be collected within each cluster. Although blinding may be difficult, the randomised timing and use of each cluster as its own control help reduce bias and address some problems caused by lack of blinding. However, this type of study design usually requires a much larger sample size (Hemming et al. 2015) and is only appropriate for certain research questions where an intervention is delivered in a group setting.

RCTs often involve strict inclusion and exclusion criteria to create a homogenous study population. This helps minimise confounding variables, factors associated with both the exposure and the outcome, that can distort the true effect of the intervention. However, this approach can limit the applicability of findings to more diverse populations, such as those with comorbidities or varying socioeconomic backgrounds (Kennedy‐Martin et al. 2015). Many RCTs exclude older participants, pregnant women, or individuals with chronic conditions, reducing the relevance of their findings to these populations (Moloney and Shiely 2022). Conducting RCT in controlled environments also limit their generalisability, as interventions are delivered under standardised conditions that may not reflect real‐world practice. Consequently, findings may not translate well across different populations and settings. While RCTs provide robust evidence with high internal validity (e.g., confidence in the cause‐effect relationship of the intervention), the limitations mentioned above can lower generalisability and external validity (Kennedy‐Martin et al. 2015), which refer to the extent to which study findings can be applied to other populations and how well the study sample represents broader groups. These challenges highlight the need for complementary research methods, such as evidence from electronic health records, to assess the broader applicability and effectiveness of interventions in a real‐world setting.

### 1.5 Electronic Health Records; A Complementary Source to Clinical Trial Data

Electronic Health Records (EHRs) are digital versions of a patient's medical history and health information (National Cancer Institute 2011). They have several advantages as sources of data over paper‐based methods for record keeping: including access to population‐level data, higher generalisability, lower attrition rates and greater statistical power (Sauer et al. 2022; Kalankesh and Monaghesh 2024). EHRs also allow researchers to analyse data concerning rare diseases that might otherwise be difficult to study in RCTs due to a low sample size. As rare conditions affect only a small number of patients, this can limit participant recruitment to clinical trials. Many of the limitations associated with RCTs can be addressed by utilising EHRs. They enable researchers to integrate health care data systems from multiple sources, potentially reducing cost and overcoming logistics challenges. EHRs allow for pragmatic trials (Angus 2015) to be incorporated into routine care. Pragmatic trials are designed to evaluate the effectiveness of interventions in real‐world settings rather than under the tightly controlled settings of more traditional RCTs (Roland and Torgerson 1998). This approach is illustrated by Hillier et al. (2021). In a study aimed to evaluate the effectiveness of two different screening strategies for GDM in women who attended antenatal clinics between 2014 and 2018 (Hillier et al. 2021). Over the 4‐year timeframe, 23 792 women were randomised to be screened for GDM by one of the two methods, and clinical outcomes compared by ITT analysis. This example demonstrates the value of pragmatic trials in implementing interventions in a real‐world clinical setting and their potential for the rapid translation of results into practice. EHRs may also address concerns associated with randomisation, particularly when different patients could benefit from alternative treatments, such as the issues mentioned above in oncology research. Thus, pragmatic trials make the research process more acceptable to both patients and clinicians.

However, these types of data do come with their own challenges (Sittig et al. 2020). At present, healthcare systems are not always able to be linked as they may not contain a unified information infrastructure (Deeny and Steventon 2015) (such as the absence of a unified participant ID or inconsistencies in the structure of collected data). There may be errors in information, the quality of the data may be poor and missing data can further impact on the overall quality. Research into EHRs has reported that individuals from minority ethnic groups are less likely to seek healthcare, leading to underrepresentation of EHR data in these cohorts (Kapadia et al. 2022). There are also data security challenges to consider as patient data must be anonymised and stored in a trusted research environment and information technology systems can fail (MIT Critical Data 2016). If such scenarios occur, sensitive patient information could be misused, potentially leading to breaches of privacy, loss of confidentiality, resulting in potential harm to the patient rather than providing a research benefit. There are also ethical considerations (Spriggs et al. 2012), as patients may not wish for their data to be used in research purposes. In England, under the NHS, medical data can be used for research, but the National data opt‐out scheme (NHS England Digital 2024) allows individuals to exclude their data from research databases. It is therefore important to engage and communicate with patients to inform and explain to them about how their data will be used. This will ensure that research databases accurately represent the population group of interest. This also allows individuals to make informed decisions regarding their data. When using EHRs as observational cohorts, residual confounding is a key limitation (Nørgaard et al. 2017) and should be accounted for in data analysis plans.

Similarly to treatment protocols in healthcare, there is not a ‘one‐size fits all approach’ when it comes to research and generating evidence. However, there are instances where electronic health records can work very effectively in certain clinical settings. For example, when data is collected routinely in a uniform manner, this enables datasets to be analysed more effectively. These scenarios include, but are not limited to, obstetric care, diabetes care and early‐life growth monitoring. For these examples there are set care pathways and data are collected in a standardised form. For instance, the early‐LIfe cross‐LInkage in Research‐Born in South London (eLIXIR‐BiSL) cohort is a partnership of data‐linkage which collects maternity, neonatal, mental health and primary care data across King's Health Partnership (Carson et al. 2020). This study has been running since 2018 and the database is expanding to incorporate health and social care data. To date, over 80 000 women and their children in the South London area have contributed data to eLIXIR‐BiSL. The study population resides in an inner‐city location and the women are of mixed ethnicity, with half of the cohort being from the global majority (i.e., of non‐white, non‐European ethnic heritage). This allows for sub‐group analyses to be performed on the data by ethnicity, without compromising on sample size. However, each population‐based cohort is unique in terms of its study population, meaning that study outcomes cannot necessarily be generalised for the UK population as a whole.

### 1.6 Electronic Health Records and Nutritional Sciences Research

As the use of EHRs has become more common, they have enabled comprehensive analyses of population level interventions. In April 2018 the UK Government mandated a soft drinks industry levy (SDIL), a public health intervention designed to encourage drinks manufacturers to reformulate drinks by reducing their sugar content, rather than be subject to a tax on drinks considered to have a high sugar content (UK Government 2016). A 2023 study using hospital level data applied the quasi‐experimental method, interrupted time series (Lopez Bernal et al. 2018), to evaluate the impact of the SDIL on hospital admissions for dental caries in children aged 0–18 years of age (Rogers et al. 2023). The findings show that over 22 months after the SDIL implementation, there was a relative reduction of 12.1% in tooth extractions for all children and the reduction for 0–4‐year‐olds was much greater at 28.6%. As mentioned above, the recent announcement by the UK Government to mandate folic acid fortification of non‐wholemeal flour (white flour) provides researchers with a unique opportunity to evaluate the impact of this intervention at a population level using birth cohorts such as eLIXIR‐BiSL. eLIXIR‐BiSL is part of the wider UK MIREDA network (Mother & Infant Research Electronic Data Analysis) (Seaborne et al. 2024), which combines routinely collected data on 100 000 antenatal and birth records annually from six UK based birth cohorts. By integrating data from these cohorts, we will be able to assess the effects of folic acid fortification of non‐wholemeal flour on a range of maternal and infant health outcomes implicated in folate insufficiency (such as neural tube defects, maternal anaemia, pre‐term birth, small‐for‐gestational age and sub‐optimal neurodevelopment) (Molloy et al. 2008; Timmermans et al. 2009). Furthermore, the scale of the data will enable an evaluation of the impact of the population level intervention across socio‐economic groups, ethnicities and geographical regions. The results from these analyses will enable researchers to identify population groups within the UK that may not fully benefit from the public health initiative. As such, the results will advise further iterations of the fortification program. This type of analysis would not be feasible or appropriate to conduct in an RCT setting due to the need for large‐scale, population‐level data and the complexities of real‐world implementation.

### 1.7 Missing Data—The Good, the Bad and the Ugly

No matter how carefully a research study is designed, some levels of missing data are inevitable. Missing data can undermine the validity and reliability of any analysis, whether in RCTs or when utilising EHRs. The presence of missing data can impact on findings and reduce statistical power. Prior to any analysis, it is important to assess the potential mechanisms behind or reasons for the missing data. The findings from this assessment will inform the rest of the analysis. Missing data have been classified into the following three types, which provide a framework for researchers to understand and address the nature of missingness (Little et al. 2012):

1. Missing Completely at Random (MCAR—The good)—the missing data are independent both of the observed and unobserved parameters. Thus, they occur entirely at random. An example of this would be that a participant fails to complete a 2‐page food frequency questionnaire as a page was missed off during the printing process.
2. Missing at Random (MAR—the bad)—the missing data are accounted for by variables recorded in the dataset. Participants from lower socio‐economic groups may be less likely to complete online dietary questionnaires due to lack of time or resources. Therefore, the missing data are related to socio‐economic status.
3. Missing Not at Random (MNAR—the ugly)—the missing data are related to the reason for the missing data. This could occur if participants who consume diets high in foods that are deemed to be unhealthy (e.g., high in sugar and fats) may be less likely to accurately report their dietary intake because they do not wish to appear to consume an ‘unhealthy’ diet. Therefore, the reason for the missing data depends on the unobserved variable itself. During the analysis of our data, we need to ensure that any analyses are not biased, and it is therefore crucial to assess mechanisms resulting in missing data and adapt analyses accordingly (Little et al. 2012).

Identifying whether data are missing randomly or due to systematic reasons can guide appropriate statistical adjustments and aid interpretation of the findings. However, this may not always be possible in EHR data. Unlike controlled research environments, where the mechanism of missingness can often be identified, data in EHRs may be missing for various reasons, such as (1) an unattended appointment, (2) variation in documentation practices by individual healthcare providers or (3) limitations in the particular EHR system (Sauer et al. 2022). This can result in bias in the findings. Therefore, it is important to report the percentage of missing data and document the demographic characteristics of those with missing data. This approach helps to contextualise why data may be missing and how this missingness impacts interpretation of findings.

## 2 Conclusion

Electronic health records offer great opportunities in the field of nutritional sciences. However, their use in research cannot replace RCTs and both research designs should complement each other. By combining both data sources (from RCTs and EHRs), researchers can address knowledge gaps, improve generalisability, analyse rare diseases, generate findings in a timelier manner and evaluate efficacy of data in real‐world settings. However, this approach can only be successful if we address the potential challenges currently faced by EHRs—data quality, data privacy and security and extent of interoperability between datasets. Collaboration between nutritional scientists and health services enables the routine inclusion of high‐quality nutrition and dietary data into datasets, helping to maximise research potential.

Furthermore, a global governing body/external research consortium should be established in order to ensure that reporting and analysis of EHRs is standardised, as this will aide in their critical appraisal and interpretation. As researchers in the field of nutritional sciences, we have the potential to support the implementation and effective use of data from EHRs in nutrition and dietetic‐related research. Key considerations regarding how we collect, manage and analyse our data need to be considered prior to data collection. We have the expertise and knowledge in this field of study and therefore should be involved in the development and optimisation of these databases. It is also imperative that we understand the limitations of our data and apply appropriate methodological approaches to present and analyse the results. While RCTs will continue to be critical for establishing causal relationships, EHRs have the potential to greatly enhance the breadth and applicability of nutrition research, thus shaping the future of the field.

## Author Contributions

Dr. Dalrymple was solely responsible for the conception, literature review, analysis and writing of this review article.

## Funding

The author has nothing to report.

## Conflicts of Interest

The author declares no conflicts of interest.

## Data Availability

The author has nothing to report.
